# Ric-8A controls Cited2 subcellular localization and transcriptional programs during neural crest development

**DOI:** 10.3389/fcell.2026.1872572

**Published:** 2026-07-15

**Authors:** Carla Castelli, Andrea Beyer, María José Ruiz, Jossef Guajardo, Lina Mariana Tovar, Juan Ignacio Leal, Ignacio Marín-Videla, Andrés Torres, Jorge Araya, Sergio Bustamante, Alejandro Maureira, José L. Gutiérrez, Estefanía Tarifeño-Saldivia, Marcela Torrejón

**Affiliations:** 1 Laboratory of Signaling and Development (LSD), Department of Biochemistry and Molecular Biology, Group for the Study of Developmental Processes (GDeP), Faculty of Biological Sciences, University of Concepción, Concepción, Chile; 2 Departamento de Ciencias Biológicas, Facultad de Ciencias de La Vida, Universidad Andres Bello, Talcahuano, Chile; 3 Department of Biochemistry and Molecular Biology, Faculty of Biological Sciences, University of Concepción, Concepción, Chile; 4 Gene Expression and Regulation Laboratory (GEaRLab), Department of Biochemistry and Molecular Biology, Faculty of Biological Sciences, University of Concepción, Concepción, Chile

**Keywords:** Cited2, development, GEF, neural crest, Ric-8A, signaling, transcriptional co-regulator

## Abstract

Neural crest (NC) cells are a highly migratory and multipotent population essential for vertebrate development, whose behavior depends on the integration of signaling pathways, transcriptional programs, and cytoskeletal dynamics. Here, we identify a previously unrecognized functional relationship between the signaling regulator Ric-8A and the transcriptional co-regulator Cited2 during NC development in *Xenopus*. Proteomic analyses and proximity ligation assays revealed Cited2 as a Ric-8A-associated factor, prompting us to investigate its role in NC biology. We show that *xtcited2* is dynamically expressed during embryogenesis, with enrichment in neural and mesodermal derivatives, including cranial NC. At the cellular level, XtCited2 exhibits a dual nuclear-cytoplasmic distribution that is regulated by XtRic-8A. Gain- and loss-of-function experiments, together with rescue assays, demonstrate that XtRic-8A controls the subcellular localization of XtCited2 and influences XtCited2-dependent transcriptional programs. Single-cell transcriptomic analyses further revealed that co-expression of *xtcited2* and *xtric-8a* defines a distinct transcriptional state enriched for genes associated with cytoskeletal organization, Rho GTPase signaling, cell migration, and cell-cycle regulation. Consistently, functional assays showed that XtRic-8A is required to maintain normal *sox2* expression, linking this regulatory axis to transcriptional control of NC identity. Together, our findings uncover a regulatory mechanism by which XtRic-8A modulates XtCited2 function, connecting protein localization, transcriptional regulation, and NC cell behavior during vertebrate development.

## Introduction

Neural crest (NC) cells are a transient, multipotent cell population unique to vertebrate embryos that arises at the neural plate border during early development. Following neural tube closure, NC cells undergo epithelial-to-mesenchymal transition (EMT), delaminate from the dorsal neural tube, and migrate extensively to generate a wide array of derivatives, including craniofacial cartilage and bone, peripheral neurons and glia, melanocytes, and key components of the cardiac outflow tract (Marcelo and de Alvcoi, 2005). Disruption of NC development leads to congenital disorders collectively known as neurocristopathies, many of which affect craniofacial and cardiac structures (Bolande, 1974; Etchevers et al., 2006; Watt and Trainor, 2014).

Neural crest (NC) cell migration is a highly dynamic and coordinated process that relies on the integration of multiple signaling pathways, transcriptional programs, and cytoskeletal remodeling mechanisms. Collective NC migration is classically regulated by contact inhibition of locomotion (CIL), co-attraction (CoA), and chemotaxis, which together ensure directional and cohesive movement (Carmona-Fontaine et al., 2008a; 2008b; 2011; Theveneau and Mayor, 2012; Roycroft and Mayor, 2016). However, this behavior is not governed solely by chemical cues. Migrating NC cells continuously interpret mechanical signals from their microenvironment, including the stiffness and architectural organization of the mesoderm-derived migratory pathways. The mechanical properties of these routes influence cell polarity, cytoskeletal dynamics, and traction force generation, thereby integrating biochemical and biomechanical inputs to fine-tune collective migration (Barriga et al., 2018). These behaviors are tightly controlled by small Rho GTPases and heterotrimeric G protein signaling, highlighting the importance of signal transduction mechanisms in NC polarity and motility (Villaseca et al., 2025; Ridley, 2015; Sah et al., 2000).

We have previously shown that Ric-8A, a non-canonical guanine nucleotide exchange factor (GEF) for Gα subunits, plays a critical role in NC migration in *Xenopus*. Ric-8A regulates cell polarity, focal adhesion dynamics, and chemotactic responses to Sdf1, and its loss of function results in defective NC migration and craniofacial abnormalities (Fuentealba et al., 2013; Toro-Tapia et al., 2017; 2018; Leal et al., 2018). These findings position Ric-8A as a key regulator of signaling pathways required for coordinated NC behavior during embryogenesis.

To further elucidate the molecular network associated with Ric-8A function, we performed a mass spectrometry-based proteomic analysis, which identified Cbp/p300-interacting transactivator, with Glu/Asp-rich carboxy-terminal domain, 2 (Cited2) as a potential interacting partner of mouse Ric-8A. Cited2 is a transcriptional co-regulator that lacks direct DNA-binding activity and modulates gene expression through interactions with multiple transcription factors (Chou et al., 2012; An et al., 2020). In mouse models, loss of *cited2* leads to early embryonic lethality and severe developmental defects, including craniofacial malformations, congenital heart defects, altered left–right patterning, and impaired migration of cardiac NC cells (Bamforth et al., 2001; 2004). In humans, *cited2* mutations have been linked to congenital heart disease and neural tube defects (Lu et al., 2010; Dianatpour et al., 2020; Chen et al., 2023).

Despite its well-established role in mammalian development, the spatiotemporal expression and function of *cited2* during early embryogenesis remain poorly characterized in *Xenopus*, particularly in the context of NC development. Given the shared involvement of Ric-8A and Cited2 in craniofacial and cardiac morphogenesis, and their potential physical interaction, we hypothesized that Cited2 may play a conserved role in NC biology. Here, we characterize the spatiotemporal expression of *cited2* during *Xenopus* development and investigate its functional role in NC cells. Using the NC as a cellular and developmental model, we explore the relationship between Cited2 and Ric-8A, providing new insights into signaling networks that regulate NC migration and that may underlie congenital craniofacial and cardiac disorders.

## Materials and methods

### Mass spectrometry analysis of mRic-8A interactors

NIH3T3 cells overexpressing c-Myc-mRic-8A or c-Myc (control) were seeded onto culture plates coated with fibronectin (5 μg/mL) and grown to approximately 60% confluence to favor cell-matrix interactions and migratory behavior. Cells were lysed in 1X phosphate-buffered saline (PBS) containing 0.5% Igepal (pH 7.4), supplemented with a protease inhibitor cocktail and 1mM PMSF. Soluble fractions containing 2mg total of protein were precleared by incubation with 40µL of Protein A/Agarose beads for 1.5 h. The precleared lysates were then incubated with 2µg of Protein A/Agarose-conjugated anti-c-Myc antibody (sc-40 AC, Santa Cruz Biotechnology) for 1.5 h. Following antibody incubation, the beads were washed four times with lysis buffer, and the immunoprecipitated protein complexes were eluted in 50 µL of 1% SDS using a cellulose acetate filter spin cup. One independent immunoprecipitation replicate was analyzed by SDS-PAGE followed by Coomassie blue staining to verify the presence of immunoprecipitated protein bands.

Samples were processed and analyzed by the GIGA-Proteomics Facility (University of Liège, Belgium) for mouse proteins identification. 5 ug of sample were reduced, alkylated and digested in solution using trypsin. Protein digests were independently analyzed by LC-ESI-MS/MS using an *Acquity* M-Class LC system (Waters) coupled to an Q Extractive ESI-orbitrap mass spectrometer (Thermo Scientific), operating in positive ion mode. Spectra were processed using Proteome Discoverer software version 2.1 (Thermo Scientific). Databases searches were performed using the Mascot server version 2.2.06 and Proteome Discoverer version 2.1 (Thermo Scientific) against the Swissprot database, restricted to *Mus musculus* taxonomy. Carbamidomethylation of Cysteine, and oxidation of methionine were set as variable modifications. A bovine serum albumin (BSA) quality control sample was digested in parallel and searched against the complete SwissProt database (all taxonomies) to monitor the efficiency and quality of the entire workflow.

### *Xenopus* fertilization and microinjection

*Xenopus* embryos were obtained by *in vitro* fertilization as previously described (Maldonado-Agurto et al., 2011; Leal et al., 2018; Toro-Tapia et al., 2018; Villaseca et al., 2025). Embryonic stages were determined according to the normal table of *Xenopus* development by Nieuwkoop and Faber (1994). For whole-mount *in situ* hybridization, wild-type *Xenopus* embryos at Nieuwkoop and Faber stages 9–45 were fixed in MEMFA solution (0.1 M MOPS, 2 mM EGTA, 1 mM MgSO_4_, 4% formaldehyde, pH 7.4) under gentle agitation for 4 h at room temperature or overnight at 4 °C. For explant assays and immunofluorescence experiments, two blastomeres of 8-cell stage *Xenopus* embryos (Nieuwkoop and Faber stage 4) were microinjected following previously established protocols (Fuentealba et al., 2013; Leal et al., 2018; Toro-Tapia et al., 2018; Villaseca et al., 2025). Each embryo was injected with either 100 pg of *ric-8a* mRNA or 15 ng of morpholino oligonucleotide (5′–GAG​GGT​ACC​CGG​ATC​CAT​GGC​TGG​C–3′; Gene Tools, Philomath, OR, United States of America), as indicated, together with 100 pg of H2B-mCherry mRNA as a lineage tracer.

### *In situ* proximity ligation assay (PLA)

To visualize the interaction between Ric-8A and Cited2 *in situ*, a Proximity Ligation Assay (PLA) was performed following a previously described protocol (Villaseca et al., 2025) with minor modifications. Briefly, neural crest (NC) cells were cultured on fibronectin-coated coverslips and fixed in 3.7% formaldehyde in PBS. Cells were permeabilized with Triton X-100, washed in PBS, and incubated in Duolink Blocking Solution (Sigma-Aldrich) for 1 h at 37 °C. Samples were subsequently incubated with primary antibodies: anti Ric-8 (Sigma, 1:25), anti-MRG1 (Santa Cruz Biotechnology, 1:25) and anti Gαi2 (Santa Cruz Biotechnology, 1:50), all diluted in Duolink Antibody Diluent (Sigma-Aldrich).

After washing with Duolink Wash Buffer A, cells were incubated with species-specific PLA PLUS and MINUS probes according to the manufacturer’s instructions (Duolink *In Situ* Detection Reagents Red, Sigma-Aldrich). Ligation and rolling-circle amplification reactions were then carried out using the reagents provided in the kit, generating fluorescent signals only when the two target proteins were located within close proximity. Following amplification, samples were washed, mounted using Duolink *In Situ* mounting medium containing DAPI, and imaged using a Leica SP8 confocal microscope equipped with a ×63 oil-immersion objective (Center for Microscopy and Advanced Bioimaging, University of Concepción). Negative controls were performed by omitting primary antibodies while maintaining all subsequent incubation and detection steps.

PLA quantification was performed by measuring the number of dots per explant and was normalized to the number of nuclei to yield the average number of puncta per cell. Additionally, the surface area of the signal was measured since potential signal clustering might prevent the resolution of individual dots.

### Cloning of cited2 cDNA and *in situ* hybridization

The coding sequence of *Xenopus tropicalis cited2* was retrieved from the NCBI database (Gene ID: 407,853) and Xenbase (XB-GENE-495035) and synthesized by Integrated DNA Technologies (IDT). XhoI restriction sites were incorporated at both ends of the sequence to facilitate downstream cloning. The synthesized fragment was subsequently ligated into the Zero Blunt™ TOPO® vector (Invitrogen) following the protocol provided by the manufacturer. The pCR-Blunt-II-TOPO/XtCited2 vector was linearized with BamHI or NotI restriction enzymes to generate antisense and sense probes, respectively. RNA probes were synthesized by *in vitro* transcription in the presence of digoxigenin-UTP using T7 RNA polymerase (antisense) or SP6 RNA polymerase (sense) from New England Biolabs, following previously described protocols (Maldonado-Agurto et al., 2011; Leal et al., 2018; Toro-Tapia et al., 2018; Villaseca et al., 2025). Probes were detected using alkaline phosphatase-conjugated anti-digoxigenin antibodies (Roche), and NBT/BCIP (nitro-blue tetrazolium chloride/5-bromo-4-chloro-3′-indolyl phosphate p-toluidine salt) was used as the chromogenic substrate. Following whole-mount *in situ* hybridization, NF stage 28 embryos were coronally sectioned at a thickness of 50 μm using a Leica VT1000M vibratome. The resulting sections were mounted in 90% glycerol. Images were acquired using a Leica S9i stereomicroscope equipped with a DFC450 camera.

### Neural crest *in vitro* culture and immunofluorescence

Neural crest (NC) explants were dissected from *Xenopus* embryos at Nieuwkoop and Faber stage 16 following previously described protocols (Fuentealba et al., 2013; Leal et al., 2018; Toro-Tapia et al., 2018; Villaseca et al., 2025). Explants were allowed to adhere to the substrate for 30 min and then permitted to migrate for 4 h. Explants were fixed in 1X DFA solution (53 mM NaCl, 10 mM Na_2_CO_3_, 4.5 mM potassium gluconate, 1 mM MgSO_4_, 1 mM CaCl_2_, 0.1% BSA, 17.5 mM bicine) supplemented with 16% formaldehyde for 20 min, followed by fixation in 1X PBS containing 3.7% formaldehyde for an additional 20 min. Samples were then washed three times for 5 min each in 1X PBS. Immunofluorescence was performed as previously described (Toro-Tapia et al., 2018) using the following antibodies: mouse anti-MRG1 (Santa Cruz Biotechnology, 1:25) and Alexa Fluor 488-conjugated anti-mouse secondary antibody (Life Technologies, 1:200). Hoechst (Thermo Fisher Scientific, 1:1000) was added together with the secondary antibody to label nuclei. After washing, samples were incubated with Phalloidin 546 (Life Technologies, 1:200) for 2 h to visualize F-actin. Images were acquired using a Leica SP8 LIGHTNING spectral confocal microscope (CMA-BioBio) University of Concepción.

### sox2 RT–qPCR analysis

Total RNA was isolated from *X*. *tropicalis* embryos at stage NF22 under different experimental conditions, including wild-type, XtRic-8A loss-of-function (Ric-8A morpholino; Ric-8AMO), and XtRic-8A overexpression (Ric-8A mRNA)-injected embryos, using the NucleoSpin RNA isolation kit (Macherey-Nagel) according to the manufacturer’s instructions. RNA integrity was assessed by electrophoresis on a 1% agarose gel, and concentration and purity were determined by spectrophotometric analysis using a NanoDrop instrument. For cDNA synthesis, 2 μg of total RNA per condition were reverse transcribed using the M-MLV Reverse Transcriptase kit (Promega), following the manufacturer’s protocol, in a final reaction volume of 25 μL and using oligo(dT) primers.

Quantitative PCR (qPCR) was performed using the Brilliant III Ultra-Fast SYBR® Green qPCR Master Mix (Agilent Technologies). Amplification was carried out under the following cycling conditions: an initial denaturation step at 95 °C for 3 min, followed by 35 cycles of denaturation at 95 °C for 30 s and annealing/extension at 60 °C for 30 s. Gene expression analysis was performed for *sox2*. Primer sequences used for amplification were as follows: pair 1(*sox2*-Forward1: 5′-CAA​CAT​GAT​GGA​GAC​CGA​TC-3′ and *sox2*-Reverse1: 5′-GCA​GAG​TGT​ACT​TAT​CCT​TCT-3′), pair 2 (*sox2*-Forward2: 5′-GTA​TGG​CAT​GAT​GCA​AGA​GC-3′ and *sox2*-Reverse2: 5′-GAA​GAA​GAG​GTG​ACT​ACA​G-3′).

### Quantification and statistical analysis

Cited2 subcellular localization was quantified from immunofluorescence images using manually segmented cell and nuclear masks. For each cell, two regions were defined: nucleus and cytoplasm (cell area excluding the nucleus). Cited2 signal was treated as a continuous variable and quantified as the mean fluorescence intensity within each region.

To reduce the impact of extreme values and standardize the dynamic range across images, intensities were percentile-normalized within the cellular mask by clipping to the 2nd-98th percentiles (p2-p98) and rescaling to [0,1]. In addition, a nuclear enrichment index (N/C) was computed per cell as N/C = mean(nucleus)/mean(cytoplasm); a small constant (e.g., 1e-6) was added to numerator and denominator to avoid division by zero. Statistical analyses used single cells as independent units. Normality of the data (normalized intensities and N/C ratios) was assessed, and statistical tests were selected accordingly. When data deviated from normality, non-parametric tests were applied. Within each condition, nucleus vs. cytoplasm intensities were compared using a paired, two-sided Wilcoxon signed-rank test. Between conditions (Wild-type, Ric-8A morphants, and Ric-8A mRNA), comparisons were performed using two-sided Mann-Whitney U. P-values were reported as ns (p ≥ 0.05), * (p < 0.05), ** (p < 0.01), and *** (p < 0.001). Radial Cited2 profiles were also computed from the nucleus toward the cell border using normalized radial distance [0,1], and reported as mean ± SEM per radial bin across cells.

### *In silico* expression analysis of cited2 and ric-8a through embryogenesis

Data analysis and figure generation were performed using Python 3.11.11. The following packages were used: Scanpy 1.11.0 (Wolf et al., 2018), NumPy 1.26.4 (Harris et al., 2020), Matplotlib 3.10.0 (Hunter, 2007), Pandas 2.2.2 (McKinney, 2010), SciPy 1.14.1 (Virtanen et al., 2020), Scikit-learn (Pedregosa et al., 2011), Statsmodels (Seabold and Perktold, 2010), and Seaborn 0.13.2 (Waskom, 2021).

Single-cell RNA sequencing (scRNA-seq) data from *X. tropicalis* embryos were obtained from the dataset reported by Petrova et al. (2024). The data were previously processed and annotated according to Nieuwkoop and Faber (NF) developmental stages (8–22) and tissue identity, and made available through an interactive online browser (Tabula *Rana*; tinyurl. com/frogatlas2). For each developmental stage, all available cells were selected. Files containing gene expression matrices, cell type annotations, and original cell indices were downloaded using the “SPRING data for selection” option within the “Download” menu. To obtain average expression values of *ric-8a* and *cited2*, cells with a relative expression level over 0 for the corresponding gene were filtered independently. To assess statistically significant differences across stages, the Shapiro–Wilk test was first applied to determine whether gene expression values at each stage followed a normal distribution. When normality was met, a Student’s t-test was performed; otherwise, the Mann–Whitney U test was used. To evaluate the correlation between *cited2* and *ric-8a* expression levels, double-positive cells (expressing both genes simultaneously) were extracted for each analyzed stage. Pearson and Spearman correlation coefficients were calculated using the stats module from SciPy.

### Differential expression analysis and functional target prediction of cited2 and ric-8a

To identify genes potentially regulated by *cited2* and *ric-8a*, all cells from developmental stage NF22 were filtered into two conditions: cells with a relative cited2 expression level over 0 and ric-8A relative expression level equal to 0 (cited2-only), and double-positive cells expressing both genes. Given the inherent scarcity of scRNA-seq datasets, where many genes are expressed in only a small fraction of cells, a consistency filter was applied to reduce noise. Genes expressed in at least 60 percent of cells in either condition were retained for downstream analysis. Differential expression analysis was then performed to identify genes whose expression levels changed in the presence of *ric-8a*. We considered a p-adjusted value of under <0.05 to determine statistical significance. Genes with a positive value of log2FoldChange were more expressed in the “Both” category in comparison to “Cited2 only”, while genes with a negative value of log2FC were more expressed in the “Cited2 only” category in comparison to the “Both” category. Gene Ontology enrichment analysis was conducted using the gProfiler web server (version e113_eg59_p19_6be52918) (Kolberg et al., 2023). Gene name abbreviations from the scRNA-seq dataset were first converted to Ensembl gene identifiers using the g:Convert tool. Manual curation was performed in cases where a single gene symbol matched multiple Ensembl IDs. The curated Ensembl IDs were subsequently used in g:GOSt to assess enrichment of Gene Ontology terms, including Molecular Function and Biological Process (Ashburner et al., 2000), as well as pathway annotations from KEGG (Kanehisa, 2000) and Reactome (Milacic et al., 2024), and Human Phenotype Ontology terms (Gargano et al., 2024). To explore potential direct targets of *cited2* and *ric-8a*, particularly in the context of NC development and migration, promoter sequence analysis was performed on genes associated with relevant GO terms. Using the BioMart application within Ensembl, upregulated genes associated with the terms signal transduction (GO:0007165), developmental process (GO:0032502), and cell motility (GO:0048870), were selected. For each selected gene, the sequences of proximal promoter, 3000 bp upstream of the transcription start site (TSS), were retrieved.

Promoter sequences were analyzed using the MEME Suite (Bailey et al., 2015). Motif discovery was first performed with MEME version 5.5.9 to identify enriched promoter motifs. Motifs with an E-value lower than 0.05 were retained. Identified motifs were then compared against the JASPAR transcription factor binding profile database using TOMTOM version 5.5.9 (Ovek Baydar et al., 2026) to identify associated transcription factors. Motifs were considered significant when E-value, p-value, and adjusted p-value were all below 0.05. A targeted literature review was subsequently conducted to evaluate potential functional links between the identified transcription factors and cited2.

## Results

### Identification of Cited2 as a Ric-8A-interacting protein

To identify proteins that interact directly or indirectly with Ric-8A, we performed an unbiased proteomic analysis based on co-immunoprecipitation followed by mass spectrometry. Proteins specifically associated with mouse Ric-8A (mRic-8A) were identified by comparison with a c-Myc control immunoprecipitation, considering both exclusive detection in the Ric-8A sample and significant enrichment relative to control based on spectral intensity (Table 1, 2).

**TABLE 1:** Mass spectrometry identification of Ric‐8A‐interacting proteins. Proteins identified following immunoprecipitation of Myc‐mRic‐8A from migrating NIH3T3 cells are listed. These correspond to proteins detected exclusively in the Myc‐mRic‐8A immunoprecipitate and not in the c‐Myc control. The accession number, protein description, peptide group score, sequence coverage (%), number of identified peptides, and area under the spectral curve are shown.

Access Code	Description	Sum PEP score	Coverage	# Peptides	Area
Q3TIR3	Synembryn-A	649.96	77.74	62	40,000,000,000
P21278	Guanine nucleotide-binding protein subunit alpha-11	79.83	31.48	10	360,000,000
P21279	Guanine nucleotide-binding protein G(q) subunit alpha	78.45	34.54	10	190,000,000
Q9DC51	Guanine nucleotide-binding protein G(k) subunit alpha	35.34	23.45	7	91,000,000
P27601	Guanine nucleotide-binding protein subunit alpha-13	33.56	23.87	8	200,000,000
P08752	Guanine nucleotide-binding protein G(i) subunit alpha-2	31.3	19.72	6	110,000,000
Q61414	Keratin, type I cytoskeletal 15	23.01	9.51	5	78,000,000
P62137	Serine/threonine-protein phosphatase PP1-alpha catalytic subunit	17.18	10.61	3	​
Q60809	CCR4-NOT transcription complex subunit 7	15.43	7.72	2	52,000,000
Q8BWD8	Cyclin-dependent kinase 19	14.15	6.99	4	15,000,000
Q9WVR4	Fragile X mental retardation syndrome-related protein 2	12.78	4.75	3	15,000,000
Q9R112	Sulfide:quinone oxidoreductase, mitochondrial	11.29	5.78	2	19,000,000
P11983	T-complex protein 1 subunit alpha	10.96	4.68	2	15,000,000
Q9JIF0	Protein arginine N-methyltransferase 1	10.39	8.63	3	56,000,000
O08664	B-cell CLL/lymphoma 7 protein family member C	9.64	10.14	2	90,000,000
Q9CQI7	U2 small nuclear ribonucleoprotein B″	8.36	11.56	2	56,000,000
P27661	Histone H2AX	6.32	22.38	2	17,000,000
Q99J72	DNA dC- > dU-editing enzyme APOBEC-3	6.2	5.13	2	59,000,000
P27600	Guanine nucleotide-binding protein subunit alpha-12	5.87	7.39	3	62,000,000
Q8BHD7	Polypyrimidine tract-binding protein 3	5.55	3.25	2	27,000,000
O35343	Importin subunit alpha-3	4.92	3.26	2	20,000,000
Q99LI9	Polyribonucleotide 5′-hydroxyl-kinase Clp1	4.77	4.24	2	24,000,000
P07724	Serum albumin	4.55	3.78	2	31,000,000
Q8CIE2	Zinc finger MIZ domain-containing protein 2	4.36	2.93	3	56,000,000

The accession number, protein description, peptide group score, sequence coverage percentage, number of identified peptides are shown and area under the spectral curve.

**TABLE 2:** Differentially enriched Ric‐8A‐interacting proteins identified by mass spectrometry. Proteins identified following immunoprecipitation of Myc‐mRic‐8A that were at least twofold more abundant in the Myc‐mRic‐8A sample than in the c‐Myc control are listed. The accession number, protein description, peptide group score, sequence coverage (%), number of identified peptides, area under the spectral curve for both the Myc‐mRic‐8A and c‐Myc samples, and the corresponding abundance ratio are shown. Cited2 (CBP/p300‐interacting transactivator 2), the interactor selected for further functional characterization in this study, is highlighted in red.

Access Code	Description	Sum PEP Score	Coverage	# Peptides	c-Myc Area	Myc-Ric-8A Area	Ratio
Q6NZC7	SEC23-interacting protein	225,83	30,46	26,00	1000000000	2100000000	2,10
P68368	Tubulin alpha-4A chain	65,59	27,23	8,00	140,000,000	290,000,000	2,07
Q9JKB3	Y-box-binding protein 3	47,94	21,61	6,00	600,000,000	1,200,000,000	2,00
Q9JKY0	CCR4-NOT transcription complex subunit 9	27,81	23,08	6,00	46,000,000	180,000,000	3,91
Q8BH59	Calcium-binding mitochondrial carrier protein Aralar1	23,61	6,79	4,00	24,000,000	53,000,000	2,21
Q64282	Interferon-induced protein with tetratricopeptide repeats 1	22,17	10,37	4,00	69,000,000	140,000,000	2,03
O35740	Cbp/p300-interacting transactivator 2	14,84	17,47	2,00	16,000,000	51,000,000	3,19
Q60716	Prolyl 4-hydroxylase subunit alpha-2	11,86	5,40	2,00	6,900,000	23,000,000	3,33
Q9D824	Pre-mRNA 3′-end-processing factor FIP1	10,41	4,30	2,00	42,000,000	110,000,000	2,62
P01633	Ig kappa chain V19-17	10,25	12,08	2,00	120,000,000	370,000,000	3,08
Q8BWW4	La-related protein 4	9,89	3,48	2,00	47,000,000	95,000,000	2,02
Q6P5E4	UDP-glucose:glycoprotein glucosyltransferase 1	9,73	2,13	2,00	13,000,000	38,000,000	2,92
Q61838	Pregnancy zone protein	8,46	1,94	3,00	19,000,000	63,000,000	3,32
Q9D338	39S ribosomal protein L19, mitochondrial	6,86	8,22	2,00	240,000,000	590,000,000	2,46

Our analysis recovered several known Ric-8A interactors, including multiple heterotrimeric G protein α subunits such as Gα13 and Gαi2 (Lin et al., 2009; Shen et al., 2012; Shen et al., 2015; Ward et al., 2015; Wiege et al., 2012; Yao et al., 2010), supporting the robustness of the approach (Table 1). Notably, among the proteins identified as enriched in Ric-8A-containing complexes, we detected Cited2 (Cbp/p300-interactive transactivator 2), a transcriptional co-regulator previously implicated in embryonic development and congenital disorders (Bamforth et al., 2001; 2004; Lu et al., 2010; Dianatpour et al., 2020; Chen et al., 2023). Cited2 was 3.19 higher abundance in the Myc-mRic-8A sample compared with the c-Myc control. For these enriched interactors, protein identification was supported by multiple quantitative parameters, including peptide score, sequence coverage, number of identified peptides, and the area under the curve of the corresponding mass spectra in both experimental and control samples, as well as the ratio between them (Table 2). The identification of Cited2 points to a potential novel association between Ric-8A and transcriptional regulatory factors, suggesting a functional link that extends beyond the well-characterized role of Ric-8A in heterotrimeric G protein signaling (Hinrichs et al., 2012).

**FIGURE 1:**
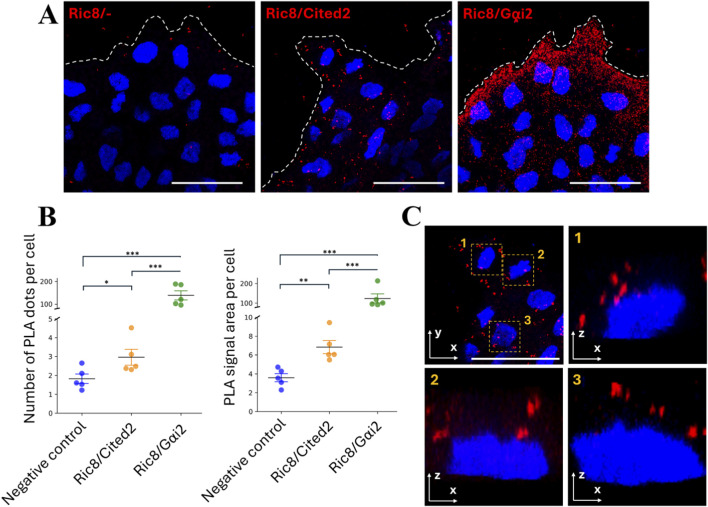
*In situ* validation of the Ric-8A/Cited2 interaction by Proximity Ligation Assay (PLA) in *Xenopus* neural crest cells. **(A)** Representative confocal images of PLA experiments performed in NC cells. PLA signals (red) indicate protein-protein interactions, and nuclei are stained with DAPI (blue). The negative control was performed in the absence of one primary antibody. The interaction between Ric-8A and Cited2 is detected as discrete PLA dots distributed throughout the cytoplasm. The previously characterized Ric-8A/Gαi2 interaction was used as a positive control. Dashed white lines delineate the borders of NC cells located at the leading edge of the explant. Scale bars, 40 μm. **(B)** Quantification of PLA signals. Left panel: number of PLA dots per cell. Right panel: total PLA-positive area per cell. Data are presented as mean ± SEM. Statistical significance was determined by one-way ANOVA followed by multiple-comparison tests (*P* < 0.05, P < 0.01, *P* < 0.001). **(C)** Three-dimensional reconstruction of PLA signals using Fiji software. Lateral views (XZ projections) of selected regions (1–3) demonstrate that Ric-8A/Cited2 PLA puncta are predominantly localized outside the nucleus, indicating that the interaction occurs mainly in the cytoplasmic compartment. Nuclei are shown in blue (DAPI) and PLA signals in red. Scale bar, 40 μm.

To independently validate the association between Ric-8A and Cited2 identified by mass spectrometry, we performed a Proximity Ligation Assay (PLA) in *Xenopus* NC cells. This approach allows the visualization of protein-protein interactions *in situ* and provides spatial information regarding the subcellular localization of the interacting partners. As shown in Figure 1A, PLA analysis revealed a clear interaction signal between Ric-8A and Cited2, supporting the existence of a physical association between these proteins in NC cells. Notably, the PLA dots were predominantly localized within the cytoplasm and were largely excluded from the nucleus (Figure 1C), suggesting that the interaction between Ric-8A and Cited2 occurs primarily in the cytoplasmic compartment.

To assess the relative strength of this interaction, we compared the Ric-8A/Cited2 PLA signal with the previously characterized Ric-8A/Gαi2 interaction, which was used as a positive control (Tall et al., 2003; Ward et al., 2015; Wiege et al., 2012; Yao et al., 2010; Chishiki et al., 2013). Although the number and area of PLA dots detected for the Ric-8A/Cited2 pair were lower than that observed for Ric-8A/Gαi2 (Figures 1A, B), a significant interaction signal was consistently detected above background levels. Interestingly, this observation is consistent with the mass spectrometry results, where Cited2 displayed a lower peptide score and area (Sum Pep Score = 14.84; area = 51,000,000) than Gαi2 (Sum Pep Score = 31.30; area = 110,000,000). Together, these findings suggest that the interaction between Ric-8A and Cited2 may be less abundant and/or more dynamic than the well-established Ric-8A/Gαi2 interaction, while nevertheless providing independent evidence supporting a specific association between Ric-8A and Cited2 *in vivo.*

Given the established roles of Ric-8A in heterotrimeric G protein signaling, cytoskeletal dynamics, and cell migration (Tall et al., 2003; Fuentealba et al., 2013; Toro-Tapia et al., 2017; 2018; Leal et al., 2018), the interaction with Cited2 suggested a potential functional connection relevant to developmental processes. This finding prompted us to further investigate the spatiotemporal expression pattern of *cited2* during *Xenopus* development and to explore its possible involvement in Ric-8A-dependent pathways during embryogenesis.

### Spatiotemporal expression analysis of *cited2* during *Xenopus* development

To characterize the spatiotemporal expression pattern of *xtcited2*, whole-mount *in situ* hybridization was performed on *Xenopus* embryos from Nieuwkoop and Faber (NF) stages 9–45. Antisense RNA probes were used to detect endogenous *xtcited2* transcripts, while sense probes served as negative controls. For each developmental stage and probe, at least 20 embryos were analyzed.

**FIGURE 2:**
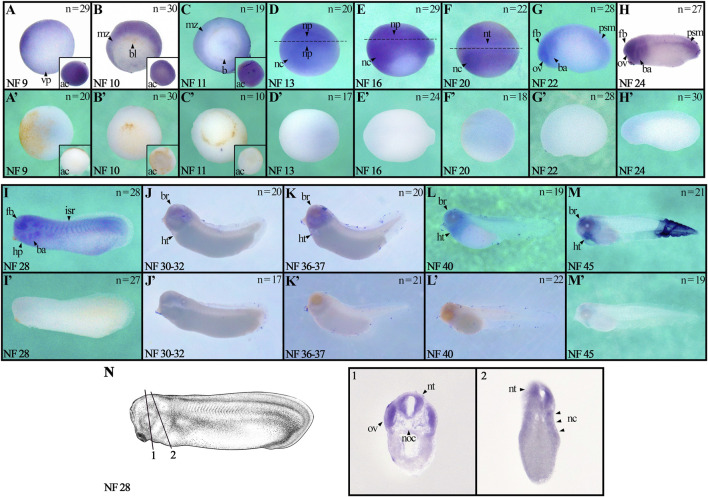
Spatiotemporal expression pattern of *cited2* during *Xenopus tropicalis* development. Whole-mount *in situ* hybridization analysis showing *cited2* expression from early embryogenesis to larval stages. From late blastula (NF9) to gastrula stages (NF11) **(A–C)** vegetal pole views are shown, with inset panels displaying animal pole views. During these stages, *cited2* expression is detected in the marginal zone and around the blastopore. From neurula (NF13) to early tailbud stages (NF24) **(D–H)** dorsal and lateral views reveal *cited2* expression in neural and mesodermal derivatives, including the neural plate, neural tube, neural crest, optic vesicle, forebrain, branchial arches, and presomitic mesoderm. During organogenesis, from tailbud (NF28) to feeding tadpole stages (NF45) **(I–M)** lateral views show sustained *cited2* expression in cranial and trunk structures, including the forebrain, brain, branchial arches, heart primordium, heart, and intersomitic regions. **(N)** Cross-section *in situ* hybridization analysis of *xtcited2* from stage NF28 *X. tropicalis* embryos. In left panels, the black lines (1 and 2) in the stage NF28 embryo indicate the position of the visualized sections. Black arrows indicate labeled anatomical structures: vp (vegetal pole), ap (animal pole), bl (blastopore lip), mz (marginal zone), b (blastopore), nc (neural crest), np (neural plate), nt (neural tube), ov (optic vesicle), fb (forebrain), ba (branchial arches), psm (pre-somitic mesoderm), hp (heart primordium), isr (intersomitic region), ht (heart), and br (brain). Control embryos hybridized with the corresponding sense RNA probe are shown in **(A’–M’)**.

At early developmental stages, *xtcited2* transcripts were broadly detected in the animal pole, with a prominent signal in the dorsal marginal zone at NF stage 10 (Figures 2A–C). During neurulation, *xtcited2* expression became enriched in neural tissues, including the neural plate, neural tube, and NC (Figures 2D–F). At NF stages 22 and 24, *xtcited2* expression was clearly observed along the migratory routes of cranial NC cells, as well as in the prospective forebrain region (Figures 2G,H). At these stages, expression was also detected in the presomitic mesoderm (Figures 2G,H).

As development progressed into organogenesis, *xtcited2* expression persisted in anterior neural structures, with strong localization in the forebrain, which later gives rise to the cerebral cortex (Figures 2I–K). In addition, *xtcited2* transcripts were detected in intersomitic regions and cardiac tissues, including the cardiac primordium (Figure 2I) and the NF 40–45 heart (Figures 2L,M). To further validate the localization of *xtcited2* transcripts observed in whole-mount embryos, vibratome transverse sections were performed at NF stage 28 (Figure 2N). These sections confirmed *xtcited2* expression in the neural tube and optic vesicle and further supported its presence along the migratory pathways of cranial NC cells. Thus, the sectional analysis corroborates the expression domains identified by whole-mount *in situ* hybridization and provides additional anatomical resolution of *xtcited2* localization during neurula and early organogenesis stages.

Interestingly, the spatiotemporal expression pattern of *xtcited2* shows notable similarities to that previously described for *xtric-8a*, as reported by Maldonado-Agurto et al. (2011) (Supplementary Figure S1; Maldonado-Agurto et al., 2011). Comparative analysis revealed that both genes are expressed in neural tissues; however, *xtcited2* displays a broader expression domain, whereas *xtric-8a* exhibits a more restricted and intense expression in specific structures such as the otic vesicle, neural tube, and their derivatives (Supplementary Figure S2; Maldonado-Agurto et al., 2011). Although *xtric-8a* expression was not detected in cardiac regions during embryonic stages, its presence has been reported in the adult *Xenopus* heart (Maldonado-Agurto et al., 2011).

Notably, while *xtric-8a* is not expressed in the presomitic mesoderm, *cited2* is prominently expressed in this region (Figure 2H), where the main interacting partners of *xtric-8a*, the α subunits of heterotrimeric G proteins, are also present (Fuentealba et al., 2016), suggesting a potential indirect functional relationship during mesodermal patterning.

### Bioinformatics analyses reveal coordinated expression of *xtcited2* and *xtric-8a* during neural crest development

To further support our experimental observations and to quantitatively assess the expression dynamics of *xtcited2* and *xtric-8a* during NC development, we performed bioinformatics analyses using publicly available single-cell transcriptomic datasets from *X. tropicalis* embryos (Petrova et al., 2024). These analyses were conducted using curated cell populations corresponding to defined developmental stages and tissue fates relevant to cranial NC formation.

**FIGURE 3:**
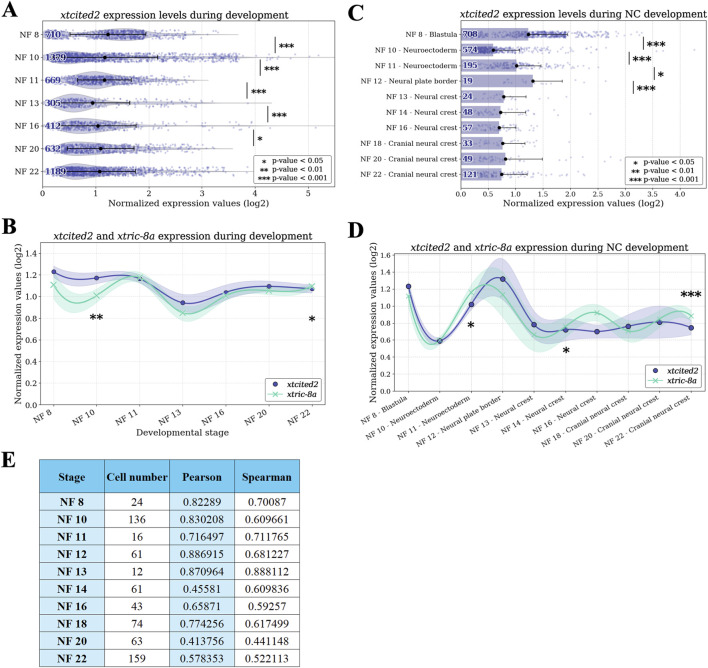
Integrated single-cell transcriptomic analysis of *cited2* and *ric-8a* expression during early *Xenopus tropicalis* development. **(A)** Single-cell RNA-seq analysis showing the developmental expression profile of *cited2* across stages corresponding to those analyzed by *in situ* hybridization (Figure 1), highlighting its dynamic regulation during early embryogenesis. **(B)** Comparative expression analysis of *cited2* and *ric-8a* across the same developmental stages reveals overlapping and stage-specific transcriptional patterns. **(C)** Spatial expression of *cited2* in tissues contributing to cranial neural crest lineages, indicating enrichment in neural and mesodermal progenitor populations. **(D)** Comparative mapping of *cited2* and *ric-8a* expression within cranial neural crest–related tissues, supporting their co-expression in relevant developmental domains. **(E)** Correlation analysis of *cited2* and *ric-8a* expression levels across embryonic stages NF8 to NF22, revealing a positive association between both genes during early development.

We first explore expression patterns at the level of whole-embryo level, by analysis all cell types by stages in the scRNAseq data. Our analysis displays that average expression levels of *xtcited2* remain relatively stable across developmental stages. However, closer inspection of data dispersion revealed significant stage-dependent differences in expression distributions, indicating dynamic regulation of *xtcited2* during development (Figure 3A). Importantly, comparison between *xtcited2* and *xtric-8a* revealed highly similar global expression patterns across stages, suggesting coordinated regulation (Figure 3B).

We next focused our analysis on cells fated to give rise to cranial NC. Within these populations, *xtcited2* exhibits high expression at the blastula stage (NF 8), followed by a marked and significant decrease upon commitment to the neuroectoderm at NF 10. Expression levels then increase significantly through the neural plate border stage (NF 12), coinciding with NC induction. Subsequently, *xtcited2* expression decreases significantly in premigratory NC cells at NF 13 and remains relatively constant through cranial NC stages up to NF 22 (Figure 3C). Analysis of *ric-8a* expression within the same cell populations revealed a broadly similar temporal profile. Direct comparison between *xtcited2*-and *xtric-8a*-expressing cells identified statistically significant differences only at specific stages, specifically in neuroectodermal cells at NF 11, NC cells at NF 16, and cranial NC cells at NF 22 (Figure 3D). Notably, both genes display their highest expression levels at stages NF11 and NF12, corresponding to NC induction and specification. This temporal overlap indicates that *xtcited2* and *xtric-8a* are expressed during the same developmental window, providing an opportunity for potential functional interactions during early NC development.

To further explore the relationship between these genes, we performed correlation analyses on cells co-expressing *xtcited*2 and *xtric-8a* across developmental stages from NF 8 to NF 22. These analyses revealed a positive correlation between *xtcited2* and *xtric-8a* expression levels across stages (Figure 3E), indicating that both genes exhibit similar expression trends during this developmental period and further supporting their presence within shared developmental contexts during cranial NC development.

### Ric-8A controls the subcellular localization of Cited2 in neural crest cells

The identification of Cited2 as a Ric-8A-associated protein prompted us to explore whether these two proteins are functionally linked during NC development. In addition to its role as a GEF for interacting Gα proteins (Tall et al., 2003), Ric-8A also functions as a molecular chaperone that stabilizes the nucleotide-free form of these proteins and is required for their proper delivery to their functional localization at the plasma membrane (Chan et al., 2013; Gabay et al., 2011; Hinrichs et al., 2012; Papasergi-Scott et al., 2018; Srivastava et al., 2019; Toro-Tapia et al., 2018). Given the role of Ric-8A in cytoskeletal dynamics and cell migration (Fuentealba et al., 2013; Toro-Tapia et al., 2018; Leal et al., 2018), together with the well-established function of Cited2 as a transcriptional co-regulator (Chou et al., 2012; An et al., 2020), we hypothesized that XtRic-8A might modulate XtCited2 activity by controlling its subcellular localization. To address this possibility, we performed immunofluorescence analyses in *Xenopus* cranial NC explants under XtRic-8A loss- and gain-of-function conditions.

Cranial NC explants were obtained from wild-type embryos, embryos microinjected with a XtRic-8A-specific morpholino to silence XtRic-8A expression (Fuentealba et al., 2013), and embryos overexpressing XtRic-8A following injection of XtRic-8A mRNA. Immunofluorescence staining was performed to visualize the subcellular distribution of XtCited2, and its localization was quantitatively assessed by measuring the nuclear-to-cytoplasmic ratio of XtCited2 signal in individual cells.

**FIGURE 4:**
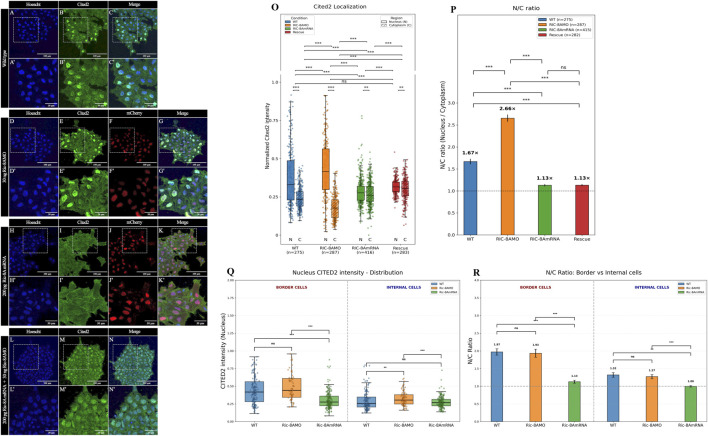
Ric-8A regulates the subcellular localization of Cited2 in neural crest cells. Wild-type explants **(A–C)** explants microinjected with 30 ng of Ric8AMO **(D–G)** explants injected with 200 pg of Ric8A mRNA **(H–K)** and rescue condition with explants injected with Ric8AMO and Ric8A mRNA **(L–N)** with corresponding higher magnification views **(A’–C’,D’–G’,H’–K’,L’–N’)** A total of 200 pg of mCherry was co-injected as a lineage tracer together with the morpholino or Ric-8A mRNA. In control explants, Cited2 localization in NC cells is predominantly nuclear, although cytoplasmic signal is also detected. Ric-8A loss of function increases the nuclear localization of Cited2, whereas Ric-8A overexpression reduces its nuclear enrichment and promotes cytoplasmic distribution. Reintroduction of Ric-8A in Ric8AMO-injected embryos reduces the nuclear accumulation of Cited2 observed in morphants, shifting its localization toward a predominantly cytoplasmic distribution. **(O)** Quantitative analysis of Cited2 immunofluorescence showing fluorescence intensity measured in the nucleus and cytoplasm under the different experimental conditions. **(P)** Nuclear-to-cytoplasmic ratio of Cited2 fluorescence intensity across the indicated conditions. **(Q)** Nuclear Cited2 fluorescence intensity in leading-edge (border) and internal cells within neural crest explants. Boxplots show the median (central line), interquartile range (box), and data range (vertical line), with individual points representing single cells. **(R)** Nuclear-to-cytoplasmic (N/C) ratio of Cited2 in leading-edge and internal cells. Bars represent mean ± SEM, and the dashed horizontal line indicates the reference value (N/C = 1). Numerical values above each bar indicate mean ratios. Statistical comparisons between conditions were performed using a two-sided Mann–Whitney U test. P values are reported as ns (p ≥ 0.05), * (p < 0.05), ** (p < 0.01), and *** (p < 0.001).

In control explants, XtCited2 displayed a mixed nuclear and cytoplasmic localization, with a balanced distribution between both compartments. In contrast, XtRic-8A knockdown resulted in a marked accumulation of XtCited2 in the nucleus, significantly increasing the nuclear-to-cytoplasmic ratio compared to control conditions (Figures 4A–G,O), with an overlap factor of N/C = 1.67 (WT) compared with N/C = 2.66 (MoXtRic-8A) (Figures 4O,P). This redistribution suggests that, in the absence of XtRic-8A, XtCited2 preferentially localizes to the nucleus.

Conversely, XtRic-8A overexpression led to a significant reduction in the nuclear pool of XtCited2, with the protein being predominantly retained in the cytoplasm (Figures 4A–C,H–K). Quantitative analysis confirmed a decrease in the nuclear-to-cytoplasmic ratio of XtCited2 in XtRic-8A-overexpressing explants relative to controls, with an overlap factor of N/C = 1.67 (WT) compared with N/C = 1.13 (mRNAXtRic-8A) (Figures 4O,P). These changes in XtCited2 localization are summarized in box plot analyses, which show a significant shift in the nuclear-to-cytoplasmic ratio of XtCited2 under XtRic-8A loss- and gain-of-function conditions compared to wild-type explants (Figure 4O). These opposing effects of XtRic-8A depletion and overexpression suggest that XtRic-8A levels tightly regulate the subcellular distribution of XtCited2.

To further validate the role of XtRic-8A in regulating XtCited2 subcellular localization, we performed rescue experiments by co-injecting XtRic-8AMO together with XtRic-8A mRNA. Under these conditions, the nuclear accumulation of XtCited2 observed in XtRic-8A morphants was markedly reduced, and XtCited2 localization shifted toward a distribution more similar to that observed in XtRic-8A-overexpressing cells (Figures 4L–O). Quantitative analysis confirmed a significant decrease in the nuclear-to-cytoplasmic ratio of XtCited2 compared with XtRic-8A morphants, indicating that reintroduction of XtRic-8A is sufficient to counteract the effects of XtRic-8A depletion on XtCited2 localization (Figures 4O,P). These rescue experiments provide additional support for the specificity of the XtRic-8AMO phenotype and further strengthen the conclusion that XtRic-8A is an important regulator of XtCited2 subcellular distribution in NC cells. Together, the loss-of-function, gain-of-function, rescue, and PLA experiments support a model in which XtRic-8A acts as a cytoplasmic regulator of XtCited2, restricting its nuclear accumulation and thereby potentially modulating the transcriptional output of XtCited2-dependent programs during NC development.

In addition to these global changes, we observed a spatial heterogeneity in XtCited2 subcellular localization within NC explants cell population. Notably, cells located at the leading edge of the explants (border cells) exhibited a higher nuclear accumulation of XtCited2, whereas cells positioned toward the interior (internal cells) displayed a more cytoplasmic distribution (Figures 4B,E,I,O). Quantitative analysis revealed that the nuclear-to-cytoplasmic ratio at the leading edge was consistently elevated, with values of 1.97 in wild-type and 1.93 in XtRic-8AMO, compared to a reduced ratio of 1.13 in XtRic-8A-mRNA (Figures 4Q,R). In contrast, cells within the interior of the explants showed lower nuclear and more homogeneous nuclear-to-cytoplasmic ratios across conditions (1.32 in wild-type, 1.27 in XtRic-8A morphants, and 1.00 in XtRic-8A-overexpressing explants) (Figures 4Q,R), closely resembling the general reduction of XtCited2-nuclear localization observed upon XtRic-8A overexpression. This subpopulation spatial organization difference is consistent with previous analyses of NC migration, where leading-edge cells exhibit increased polarity and migratory activity compared to inner cell populations (Carmona-Fontaine et al., 2008a; Theveneau et al., 2010; Toro-Tapia et al., 2018; Villaseca et al., 2025). These observations suggest that XtCited2 nuclear localization is enriched in more active and polarized cells at the migratory front, supporting a potential role for this transcriptional regulator in modulating cellular states associated with enhanced migratory behavior.

### *In silico* analysis of Xtcited2-XtRic-8A functional interplay at the single-cell level

Our *in vivo* and *ex vivo* analyses indicate that XtRic-8A modulates the subcellular distribution of XtCited2 in NC cells, suggesting a functional interplay that may influence XtCited2-dependent transcriptional programs. To determine whether this interaction is reflected at the transcriptional level *in vivo*, we focused our analysis on stage NF22 using the previously described publicly available single-cell transcriptomic dataset (Petrova et al., 2024). This developmental stage was selected because it contains the highest number of cells co-expressing *xtcited2* and *xtric-8a* (Figure 3E) and corresponds to a period when cranial NC cells are actively migrating.

We focused our analysis on *xtcited2*-expressing cells and stratified them into two populations based on the presence or absence of *xtric-8a* expression, generating *xtcited2* ^+^/*xtric-8a* ^+^ (double-positive) and *xtcited2* ^+^/*xtric-8a* ^−^ cell groups. Among the 45,786 cells analyzed at NF stage 22, these 2 cell populations accounted for 2.25% and 0.35% of the total cell population, respectively. They encompassed diverse cell types, predominantly of neural and mesodermal origin, as illustrated in Supplementary Figure S3 (Supplementary material). To reduce noise associated with lowly expressed transcripts, we retained only genes expressed in at least 60% of cells in either category. Using this filtered gene set, we performed differential expression analysis to identify transcriptional differences associated with the presence of *xtric-8a*.

**FIGURE 5:**
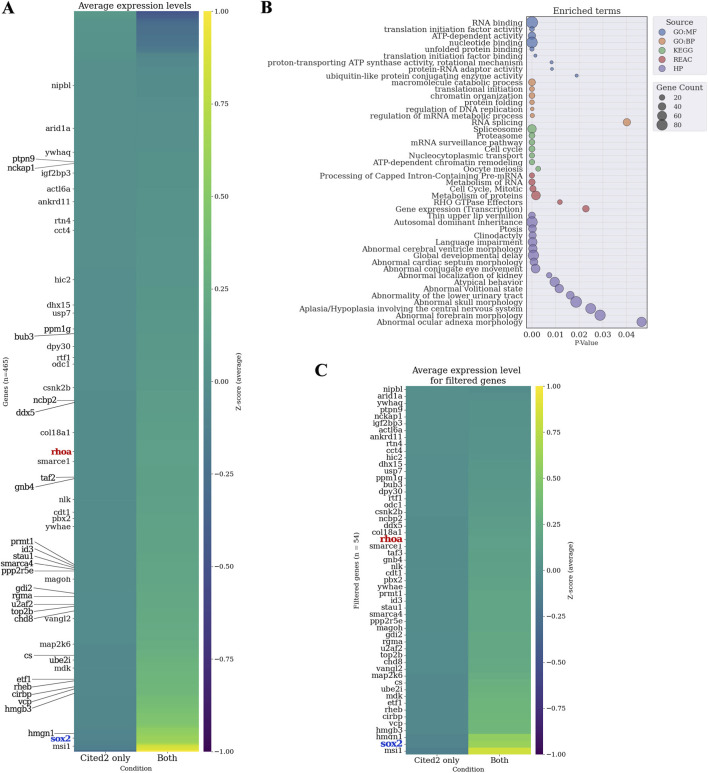
Differential gene expression, functional enrichment, and experimental validation of Sox2 expression in *xtcited2* ^*+*^ cell populations with or without *xtric-8a*. **(A)** Heatmap showing genes with significantly differential expression between cells expressing *cited2* alone and cells co-expressing *cited2* and *ric8a*. **(B)** Enrichment analysis of functional categories identified among the differentially expressed genes, including Molecular Function (blue), Biological Process (orange), KEGG pathways (green), Reactome pathways (red), and Human Phenotype Ontology terms (purple). Circle size represents the number of genes associated with each biological term, as indicated in the legend. **(C)** Heatmap showing the expression of selected genes in “Cited2-only” and “Both” cell populations associated with Gene Ontology terms related to cell motility, signal transduction, and developmental processes, revealing distinct transcriptional profiles between groups. Genes displayed in the heatmap were selected based on an adjusted p value (p-adj) < 0.05.

This analysis revealed that the vast majority of the 465 differentially expressed genes were upregulated in *xtcited2* ^+^/*xtric-8a* ^+^ cells, indicating that these genes are likely positively regulated in the context of combined *xtcited2* and *xtric-8a* expression. Accordingly, subsequent analyses focused on this positively regulated gene subset. Heatmap visualization of gene expression profiles revealed that, while most genes displayed intermediate expression levels, the two populations exhibited distinct transcriptional signatures (Figure 5). Notably, and in the context of NC, the transcription factor *sox2* and the small GTPase *rhoa* emerged as prominently upregulated genes in the double-positive population (Figure 5A).

*Sox2* is a key transcription factor in NC development and has been closely associated with the maintenance of mesenchymal identity and stemness in embryonic stem cells (ESCs). In mouse ESCs, *cited2* overexpression has been shown to enhance *sox2* expression in the context of stemness maintenance (Li et al., 2012), whereas loss of *cited2* delays *sox2* silencing during cardiomyocyte differentiation (Kranc et al., 2015). More recently, the transcription factor TFAP2C, a known Cited2 interactor, was shown to regulate *sox2* expression during pre-implantation development (Bragança et al., 2003; Kim et al., 2025), further supporting a functional link between Cited2 and *sox2* regulation.

*rhoa* encodes a small GTPase that plays a central role in cytoskeletal remodeling during NC migration. Its expression is regulated by both HIF-1α and the Myc–Skp2–Miz1–p300 transcriptional complex (Chan et al., 2010; Kim et al., 2018). Given that Cited2 functions as a competitive inhibitor of HIF-1α and interacts with Myc and p300, it is plausible that Cited2 modulates *rhoa* expression through similar regulatory pathways (An et al., 2020; Bhattacharya et al., 2001; Freedman et al., 2003).

Pathway enrichment analysis of genes upregulated in *xtcited2* ^+^/*xtric-8a* ^+^ cells revealed significant enrichment of pathways associated with post-transcriptional regulation, protein degradation, translation initiation, and cell cycle control (Figure 5B). In particular, increased activity of spliceosome- and proteasome-related pathways, as well as chromatin remodeling processes, suggests that these cells may be undergoing active proliferation or differentiation (Ashburner et al., 2000; Kanehisa, 2000; Milacic et al., 2024). Strikingly, enrichment of Rho GTPase effector pathways was also observed, consistent with potential cytoskeletal and structural changes within these cells (Fuentealba et al., 2013; Toro-Tapia et al., 2018; Leal et al., 2018). Moreover, the upregulated gene set was associated with human phenotypes related to developmental defects, including abnormalities in brain, cardiac, and urinary system morphogenesis, as well as global developmental delay (Human phenotype ontology (Gargano et al., 2024) (Figure 5B).

**FIGURE 6:**
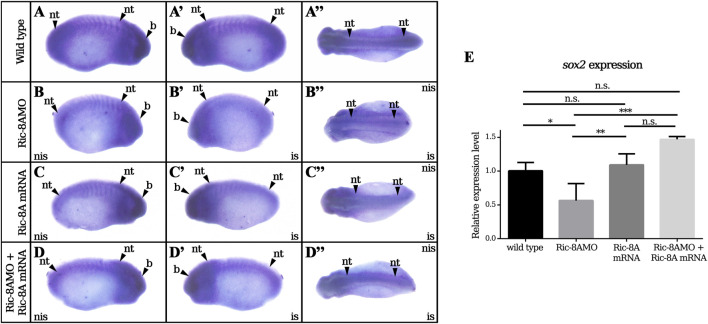
Validation of *sox2* expression changes associated with the Ric-8A/Cited2 regulatory pathway during *Xenopus* embryonic development. Right lateral **(A–D)**, left lateral **(A’–D’)**, and dorsal **(A”–D”)** views of stage NF22 *Xenopus* embryos are shown. Wild-type embryos **(A,A’,A”)** display *sox2* expression in the neural tissue and intersomitic region. On the injected side of Ric-8A morphants **(B’)** *sox2* expression is absent from the intersomitic region, whereas co-injection of Ric-8A mRNA restores this expression pattern **(D’)** No obvious differences in *sox2* expression were observed between wild-type embryos and embryos overexpressing Ric-8A **(C’) (E)** RT-qPCR analysis of *sox2* expression levels under Ric-8A knockdown, overexpression, and rescue conditions. *sox2* expression was significantly reduced in Ric-8A morphants, whereas a slight, non-significant increase was observed in Ric-8A-overexpressing embryos compared with controls. In the rescue condition, *sox2* expression was significantly increased relative to the knockdown condition, approaching control levels. Abbreviations: isr, intersomitic region; nt, neural tube; ov, optic vesicle; nis, non-injected side; is, injected side.

To experimentally validate the transcriptional trends observed in the single-cell transcriptomic analysis, we assessed *sox2* expression *in vivo* under conditions of xtRic-8A loss- and gain-of-function using both whole-mount *in situ* hybridization and RT-qPCR. For the *in situ* analyses, *X. tropicalis* embryos were examined at stage NF22 under wild-type, xtRic-8A knockdown, xtRic-8A overexpression, and rescue conditions. In wild-type embryos, as expected *sox2* expression was detected in the brain and neural tube regions (Figures 6A–A”). Upon xtRic-8A knockdown, a slight reduction in *sox2* expression was observed on the injected side of the embryo (Figures 6B–B”). In contrast, embryos overexpressing xtRic-8A did not display obvious changes in the spatial pattern or intensity of *sox2* expression compared with controls (Figures 6C–C”). Importantly, co-injection of xtRic-8AMO together with xtRic-8A mRNA restored *sox2* expression toward wild-type phenotype (Figures 6D–D”), providing additional evidence for the specificity of the xtRic-8AMO phenotype.

To complement these observations, total RNA was extracted from *X. tropicalis* embryos at stage NF22, and *sox2* expression was quantified by RT-qPCR. Consistent with both the *in situ* hybridization results and the *in silico* analysis, *sox2* expression was significantly reduced upon xtRic-8A depletion compared with wild-type embryos, resembling the transcriptional profile observed in xtcited2^+^/xtric-8a^−^ cell groups expressing *xtcited2* in the absence of *xtric-8a* (Figure 6E). In contrast, no significant differences in *sox2* expression were detected between wild-type and xtRic-8A overexpression conditions, although a consistent upward trend was observed across biological replicates (Figure 6E). To further evaluate the specificity of the observed transcriptional effects, we performed rescue experiments by co-injecting xtRic-8AMO together with xtRic-8A mRNA and subsequently assessing *sox2* expression by RT-qPCR. Reintroduction of xtRic-8A restored *sox2* expression levels to values comparable to those observed in wild-type embryos (Figure 6E), demonstrating that the reduction in *sox2* expression observed in xtRic-8A morphants is specifically associated with the loss of xtRic-8A function.

Together, these findings support a role for xtRic-8A in the maintenance of normal *sox2* mRNA levels during embryonic development, provide experimental validation of transcriptional changes predicted from the single-cell transcriptomic analysis, suggest a functional relationship between Ric-8A and Cited2-associated transcriptional programs, and further validate the specificity of the xtRic-8AMO-induced phenotype through rescue of *sox2* expression.

To explore potential transcriptional regulators associated with the gene expression changes identified in NC cells, we performed a predictive promoter motif enrichment analysis using a subset of 54 significantly upregulated genes associated with cell motility, signal transduction, and developmental processes (Figure 5C; Supplementary Figure S4A). This analysis identified 22 significantly enriched motifs, which were subsequently compared against the JASPAR transcription factor database (Ovek Baydar et al., 2026). Among the enriched motifs, high-confidence matches were identified for Znf281, Znf740, and Znf148, and binding sites for these transcription factors were detected in the promoters of genes such as *sox2* and *rhoa* (Supplementary Figure S4B). In addition, binding motifs for Sp1 were identified in the *sox2* promoter. These findings suggest potential transcriptional regulatory networks that may be associated with the transcriptional changes observed in cells co-expressing *cited2* and *ric-8A*. However, as this analysis is based exclusively on bioinformatic predictions and no functional validation was performed, these results should be considered as candidate regulatory mechanisms that warrant future experimental investigation.

Collectively, these findings support a functional interplay between XtRic-8A and XtCited2 and suggest that XtRic-8A contributes to the regulation of XtCited2-dependent transcriptional programs during NC development.

## Discussion

The development of NC cells depends on a tight coordination between transcriptional regulation and dynamic cellular processes such as epithelial-to-mesenchymal transition, collective migration, and extensive remodeling of the cytoskeleton (Toro-Tapia et al., 2017; Rogers et al., 2012). Disruption of these processes underlies a broad spectrum of developmental disorders collectively known as neurocristopathies (Rajan and Hutchins, 2024). In line with this, Ric-8A has been shown to regulate cytoskeletal organization, thereby controlling embryogenesis and NC cell migration (Fuentealba et al., 2013; Toro-Tapia et al., 2018; Leal et al., 2018; Tõnissoo et al., 2006). In this study, we identify a previously unrecognized functional relationship between the transcriptional co-regulator Cited2 and the signaling regulator Ric-8A, and propose that their interplay contributes to the regulation of NC developmental programs in *Xenopus*.

Our spatiotemporal analysis establishes that *xtcited2* is dynamically expressed during *Xenopus* embryogenesis, with highest levels during early developmental stages and a pronounced enrichment in neural and mesodermal derivatives, including cranial NC. This expression profile is consistent with bulk and single-cell RNA-seq datasets from *Xenopus* (Owens et al., 2016; Session et al., 2016) and resembles *cited2* expression patterns reported in mouse, chick, and human embryos, where Cited2 is associated with neural, cardiac, and mesenchymal tissues (Bhattacharya et al., 1999; Dunwoodie et al., 1998; Schlange et al., 2000; Bhattacherjee et al., 2009). Together, these observations support a conserved role for Cited2 in NC-derived and related tissues. Furthermore, vibratome sections confirmed the expression domains identified by whole-mount *in situ* hybridization, providing additional anatomical support for *xtcited2* expression in the neural tube, optic vesicle, and cranial NC migratory pathways.

A central finding of this study is the identification of Cited2 as a previously unrecognized Ric-8A-associated factor. Cited2 was initially identified through proteomic analysis as a candidate Ric-8A-interacting protein and this association was further supported by proximity ligation assays in NC cells. Although PLA does not demonstrate direct physical binding, the presence of PLA-positive dots indicates that both proteins are located in close proximity *in vivo* and likely participate in a common molecular complex. Together, these complementary approaches provide independent lines of evidence supporting a functional association between XtRic-8A and XtCited2 during NC development. At the cellular level, we observed that XtCited2 displays a dual nuclear-cytoplasmic localization in *Xenopu*s cranial NC cells, consistent with previous reports in mammalian cell lines (Thul et al., 2017). Given the established role of Cited2 as a transcriptional co-regulator, its nuclear localization is expected; however, the presence of a detectable cytoplasmic pool suggests an additional layer of regulation. Importantly, our functional experiments demonstrate that perturbation of XtRic-8A levels significantly alters the nuclear-to-cytoplasmic distribution ratio of XtCited2, providing the first *ex vivo* evidence that XtRic-8A regulates XtCited2 subcellular localization. Moreover, the altered localization observed following morpholino-mediated depletion of XtRic-8A was restored by re-expression of XtRic-8A, supporting the specificity of the knockdown phenotype and reinforcing the conclusion that XtRic-8A contributes to the regulation of XtCited2 localization in NC cells.

Based on these observations, we propose a model in which XtRic-8A modulates the availability of XtCited2 for nuclear functions. The increased nuclear accumulation of XtCited2 observed following XtRic-8A depletion, together with the stronger reduction of the nuclear localization observed under XtRic-8A overexpression conditions, is consistent with a role for XtRic-8A in regulating the balance between cytoplasmic and nuclear pools of XtCited2. However, the molecular mechanisms underlying this regulation remain unclear. Indeed, the effects of XtRic-8A on XtCited2 localization may extend beyond a simple retention mechanism. Ric-8A has been reported to function as a molecular chaperone that stabilizes Gα proteins and protects them from proteasomal degradation (Klattenhoff et al., 2003; Jenie et al., 2013; Gabay et al., 2011). Interestingly, Cited2 itself is regulated through ubiquitin-proteasome-dependent pathways (Shin et al., 2008). Therefore, it is conceivable that XtRic-8A influences not only the subcellular localization of XtCited2 but also its stability and turnover. This possibility may help explain why changes in XtRic-8A levels strongly affect XtCited2 localization while not necessarily producing proportional changes in downstream transcriptional outputs. In this context, measuring *xtcited2* transcript levels may provide a cleaner readout of transcriptional regulation than protein abundance, which could be influenced by additional post-transcriptional and post-translational mechanisms. Future studies will be required to determine whether XtRic-8A regulates XtCited2 through cytoplasmic retention, protein stabilization, control of nuclear trafficking, or a combination of these mechanisms.

The spatial enrichment of nuclear XtCited2 at the leading edge of NC explants suggests that its activity may be associated with highly dynamic and polarized cellular states. This observation extends the potential relevance of Cited2 beyond developmental patterning and aligns with its established roles in proliferation, survival, cellular plasticity, and loss of contact inhibition in cancer cells (An et al., 2020). In this context, the preferential nuclear localization of XtCited2 in leading-edge cells may reflect a conserved role in promoting transcriptional programs associated with cellular responsiveness and migratory behavior. Thus, the XtRic-8A-XtCited2 axis may represent a regulatory module that coordinates NC cell behavior during development while sharing mechanistic similarities with pathways co-opted during cancer progression. The relevance of this regulatory axis is further reinforced by our single-cell transcriptomic analyses. However, interpretation of these datasets requires consideration of the distinct experimental contexts used throughout this study. The single-cell transcriptomic analysis reflects endogenous transcriptional states in wild-type embryos and compares cell populations that naturally express or do not express *xtric-8a*. In contrast, our functional studies relied on morpholino-mediated depletion, which produces a knockdown rather than a complete loss of function. Furthermore, RT-qPCR analyses were performed using whole embryos, whereas the localization studies were conducted specifically in NC cells. These differences in biological resolution are likely to contribute to the distinct magnitudes of the effects observed across experimental approaches.

By comparing *xtcited2+/xtric-8a−* and *xtcited2+/xtric-8a +* cell populations at stage NF22, a stage characterized by active cranial NC migration, we identified distinct transcriptional states associated with *xtric-8a* expression. Cells co-expressing both genes displayed enrichment of pathways related to cytoskeletal organization, Rho GTPase signaling, cell-cycle regulation, and post-transcriptional control. These processes are intimately linked to NC migratory behavior and support the notion that the XtRic-8A-XtCited2 axis influences functional cellular states rather than isolated transcriptional events. Importantly, these biological processes emerged from ontology analyses of genes enriched in the *xtcited2+/xtric-8a +* population, indicating that the presence of *xtric-8a* is associated with a distinct transcriptional state characterized by programs linked to cytoskeletal organization, cell migration, proliferation, and gene regulatory control. This observation is consistent with the proposed role of XtRic-8A as a modulator of NC cellular states rather than a regulator of individual target genes.

Among the differentially expressed genes, *sox2* emerged as a particularly compelling candidate. Sox2 has been implicated in NC competence and maintenance of stem-like cellular states, and previous studies have shown that its expression can be influenced by Cited2-dependent transcriptional mechanisms involving TFAP2 family members (Bragança et al., 2003; Li et al., 2012; Kranc et al., 2015; Kim et al., 2025). Consistent with this model, we found that *sox2* mRNA levels were significantly reduced following xtRic-8A depletion, recapitulating the transcriptional profile observed in cells expressing *xtcited2* in the absence of *xtric-8a*. Importantly, this phenotype was rescued by reintroduction of *xtRic-8A*, both in whole-mount *in situ* hybridization and RT-qPCR experiments, supporting the specificity of the observed effect and providing *in vivo* evidence that XtRic-8A contributes to the maintenance of normal *sox2* mRNA levels.

Interestingly, the marked reduction in nuclear XtCited2 localization induced by XtRic-8A overexpression was not accompanied by comparable changes in *sox2* mRNA expression. This apparent discrepancy may reflect the distinct biological scales captured by each assay. Whereas XtCited2 localization was examined specifically in NC cells, *sox2* RT-qPCR measurements represent an average across all embryonic tissues and cell populations. Consequently, transcriptional responses occurring in a relatively small subset of cells may become diluted when analyzed at the whole-embryo level. In addition, the transcriptional activity of Cited2 is likely influenced by the availability of cell type-specific transcriptional partners (Bhattacharya et al., 2001; Shin et al., 2018) and chromatin context (Liu et al., 2015), suggesting that changes in nuclear localization may not necessarily translate into proportional changes in target gene expression (Chou et al., 2012). Nevertheless, the rescue of both the localization and *sox2* expression phenotypes strongly supports a functional relationship between XtRic-8A and XtCited2.

RhoA emerged as another candidate downstream of the XtRic-8A-XtCited2 axis from our single-cell transcriptomic analysis, where *rhoa* expression was enriched in the cell population co-expressing *xtcited2* and *xtric-8a*. Given its well-established role as a central regulator of cytoskeletal dynamics, cell morphology, and NC migration (Jaffe and Hall, 2005), *rhoa* represented an attractive candidate linking the transcriptional changes identified in our analysis to NC cell behavior. However, unlike *sox2*, we did not detect significant changes in *rhoa* mRNA levels in our whole-embryo RT-qPCR experiments following manipulation of xtRic-8A expression (data not shown). One possible explanation for this apparent discrepancy is that *rhoa* expression may be subject to multiple compensatory regulatory mechanisms. Previous studies have shown that Cited2 can functionally interact with HIF-dependent transcriptional programs (Qin et al., 2021), while HIF activity positively regulates *rhoa* expression (Du et al., 2025). Conversely, the Protein Arginine Methyltransferase 1 (PRMT1) has been described as a negative regulator of HIF activity (Lafleur et al., 2014). Interestingly, our single-cell transcriptomic analysis revealed increased PRMT1 expression in the cell population co-expressing *xtric-8a* and *xtcited2*. Thus, opposing regulatory inputs involving Cited2/HIF and PRMT1/HIF may contribute to maintaining *rhoa* expression levels, potentially buffering transcriptional changes that might otherwise be expected. Furthermore, such effects could be obscured by the heterogeneous cellular populations represented in whole-embryo RT-qPCR analyses, in contrast to the cell-type-specific resolution provided by single-cell transcriptomics. At present, however, any mechanistic relationship between XtRic-8A, XtCited2, and RhoA remains speculative and will require direct experimental validation.

Beyond *sox2* and *rhoa*, numerous additional genes were differentially expressed in the *xtcited2+/xtric-8a +* cell population, suggesting that the functional impact of this regulatory axis extends beyond the pathways explored here. Likewise, promoter motif enrichment analyses identified candidate transcription factor families potentially associated with genes upregulated in these cells, including factors linked to stemness, epithelial-to-mesenchymal transition, and transcriptional co-regulation like ZNF281 (Hahn and Hermeking, 2014; Hahn and Hermeking, 2014; Kranc et al., 2015; Li et al., 2012). While these analyses do not establish direct regulatory relationships, they provide a valuable framework for future studies aimed at identifying the transcriptional partners and regulatory networks through which XtRic-8A and XtCited2 influence NC development.

**FIGURE 7:**
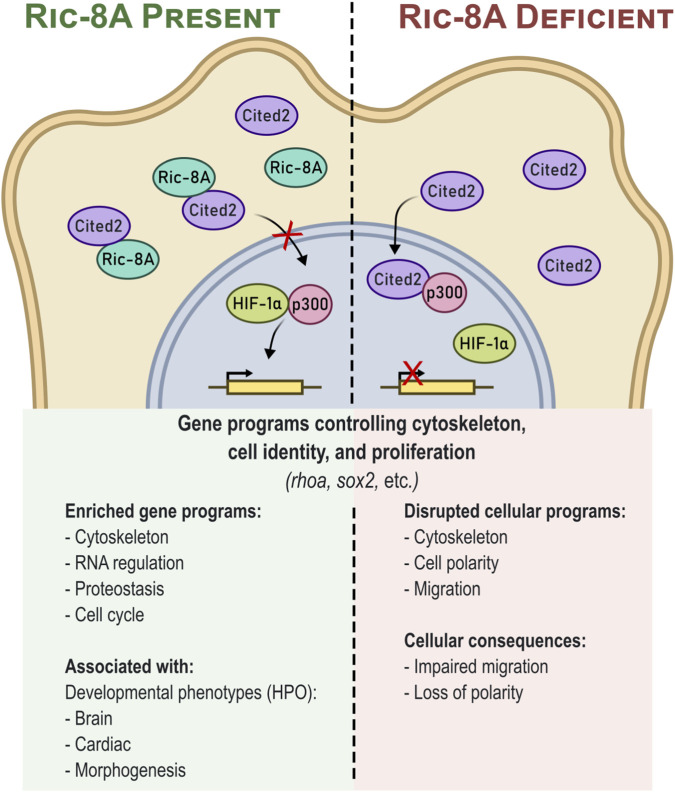
Model of XtRic-8A-dependent regulation of XtCited2 localization and transcriptional programs in neural crest cells. Schematic representation of how XtRic-8A modulates XtCited2 subcellular distribution and downstream gene expression. In the presence of XtRic-8A (left), XtCited2 is partially retained in the cytoplasm, limiting its nuclear availability and enabling balanced transcriptional activity in cooperation with nuclear partners such as p300 and HIF-1α. This condition is associated with enrichment of gene programs related to cytoskeletal organization, RNA regulation, proteostasis, and cell cycle, including genes such as *rhoa* and *sox2*. These transcriptional programs are linked to developmental phenotypes, including brain, cardiac, and morphogenetic processes. In contrast, under XtRic-8A-deficient conditions (right), XtCited2 accumulates in the nucleus, leading to altered transcriptional output and disruption of key cellular programs. This imbalance is associated with defects in cytoskeletal organization, loss of cell polarity, and impaired migration. The model distinguishes between intracellular regulatory mechanisms and their broader biological associations, highlighting the role of the XtRic-8A–XtCited2 axis in coordinating neural crest cell behavior.

From a broader perspective, our data position the XtRic-8A-XtCited2 axis as a regulatory interface connecting signaling pathways, protein dynamics, and transcriptional programs during NC development. Given the association of both proteins with congenital heart defects, craniofacial abnormalities, and pathological cell migration (Bamforth et al., 2001; 2004; Yin et al., 2002; MacDonald et al., 2008; Fuentealba et al., 2013), this mechanism may have implications beyond embryogenesis. In particular, dysregulation of XtCited2 localization or XtRic-8A function could contribute to disease states characterized by altered cell migration, including cancer invasion and fibrotic remodeling (Figure 7).

In summary, our study identifies XtCited2 as a novel XtRic-8A-associated factor and supports a model in which XtRic-8A regulates XtCited2-dependent transcriptional programs by controlling its subcellular localization. By integrating expression analyses, proximity assays, rescue experiments, *ex vivo* functional studies, and single-cell transcriptomics, we provide a multiscale framework linking protein dynamics to gene regulation and cell behavior (Figure 7). These findings establish a foundation for future studies aimed at dissecting how signaling regulators and transcriptional co-regulators cooperate to control NC development and its associated pathologies.

## Conclusion

In conclusion, our findings identify XtRic-8A as a previously unrecognized modulator of XtCited2 function during NC development. The overlapping expression patterns of both factors, together with proximity assays, functional perturbation experiments, rescue analyses, and single-cell transcriptomic profiling, support a model in which XtRic-8A regulates the activity of XtCited2 by controlling its subcellular localization. This regulation is associated with distinct transcriptional states and influences NC-associated gene expression programs, including the maintenance of *sox2* expression. By linking the spatial regulation of a transcriptional co-regulator to gene expression and NC cell behavior, our study provides new insight into how signaling regulators and transcriptional networks cooperate to coordinate embryonic development and establishes a framework for future studies addressing the molecular mechanisms underlying this regulatory axis.

## Data Availability

The original contributions presented in the study are included in the article/Supplementary Material, further inquiries can be directed to the corresponding authors.

## Supplementary material

SUPPLEMENTARY FIGURE S1 Comparison of *xtcited2* and *xtric-8a* expression patterns during early development and neurulation in *Xenopus tropicalis*. Both *xtcited2* (top) and *xtric-8a* (bottom) are expressed in the marginal zone and in neural tissues, including the neural tube and neural crest, as well as in the ocular region. From late neurula stages onwards, expression is also observed in brain regions, the optic domain, and the presomitic mesoderm. (Top panels) Black arrowheads indicate specific structures labeled as follows: vp (vegetal pole), ac (animal cap), bl (blastopore lip), mz (marginal zone), b (blastopore), nc (neural crest), np (neural plate), nt (neural tube), ov (optic vesicle), fb (forebrain), ba (branchial arches), psm (presomitic mesoderm). (Bottom panels) Red arrows indicate specific structures labeled as follows: mz (marginal zone), bl (blastopore lip), ppe (pre-placodal ectoderm), r (rhombomeres), nc (neural crest), nt (neural tube). Modified from Maldonado-Agurto et al., 2011.

SUPPLEMENTARY FIGURE S2 Comparison of *xtcited2* and *xtric-8a* expression patterns during organogenesis in *Xenopus tropicalis*. The expression of *xtcited2* becomes more restricted to cephalic tissues, including the forebrain, midbrain, and hindbrain. Between NF30 and 37, *xtcited2* is predominantly expressed in the cranial region, whereas *xtric-8a* is observed in optic and neural tissues. From NF40 onwards, *xtcited2* expression is also detected in the developing heart. (Top panels) Black arrowheads indicate specific structures labeled as follows: fb (forebrain), ba (branchial arches), hp (heart primordium), isr (intersomitic region), ht (heart), and br (brain). (Bottom panels) Red arrows indicate specific structures labeled as follows: mhb (mid-hindbrain boundary), fb (forebrain), b (brain), oc (otic capsule), opc (optic cup), ba (branchial arches), nt (neural tube), pg (pineal gland), and rl (rhombic lip). Modified from Maldonado-Agurto et al., 2011.

SUPPLEMENTARY FIGURE S3 Distribution of cell types in cited2-expressing and double-positive cell populations. Bar plots showing the distribution of cell types among cells expressing cited2 only (1,030 cells) and double-positive cells (159 cells), respectively. Cell populations are color-coded by tissue identity, and the percentage of cells belonging to each cell group is indicated according to the legend on the left. **(A)** Cells expressing cited2 alone are predominantly of mesodermal and neural identity, whereas double-positive cells are mainly of neural origin. For each population, cells expressing *sox2* **(B)** or *rhoa* **(C)** are further classified according to tissue identity. Gray bars indicate the percentage of cells that do not express the corresponding gene. As expected, *sox2* expression is largely restricted to neural cell populations, whereas *rhoa* is broadly expressed across nearly all analyzed cell types.

SUPPLEMENTARY FIGURE S4 Gene ontology-associated gene sets and promoter motif enrichment analysis. **(A)** Genes associated with selected Gene Ontology terms, including cell motility, signal transduction, and developmental processes, providing functional context for the transcriptional programs identified. **(B)** Summary of MEME analysis results. Motif discovery performed on the 5′regulatory regions of selected genes identified 22 enriched motifs, of which three showed significant matches to known transcription factor binding sites in the JASPAR database based on p-value and E-value criteria. These motifs are associated with candidate transcription factors potentially regulating the observed gene expression patterns.
