# Supplementary figures and images for: Ric-8A controls Cited2 subcellular localization and transcriptional programs during neural crest development
Source: Front Cell Dev Biol. 2026 Jul 15;14:1872572. doi: 10.3389/fcell.2026.1872572 (PMC13416619; doi:10.3389/fcell.2026.1872572)

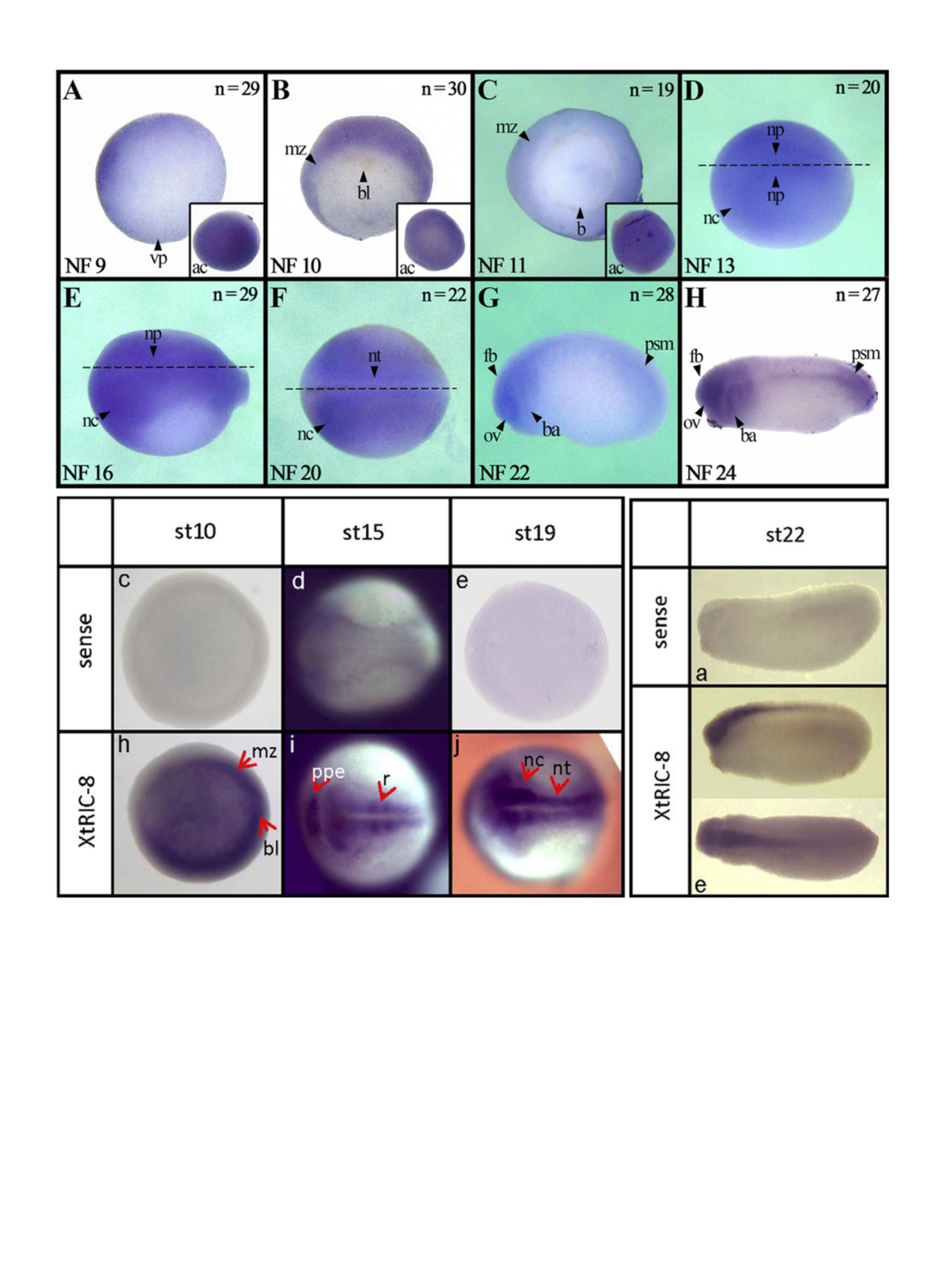

Supplement: Supplementary file 1 [file Image1.tiff]

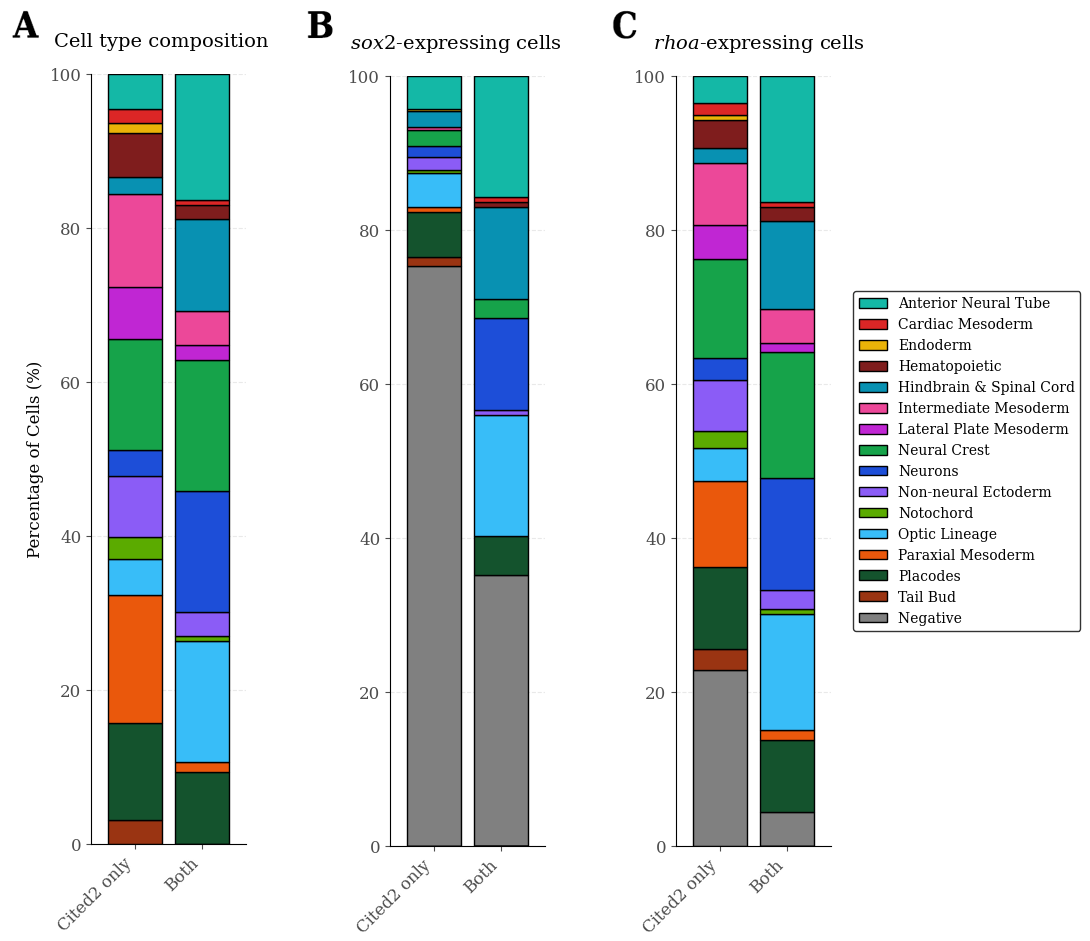

Supplement: Supplementary file 2 [file Image3.tif]

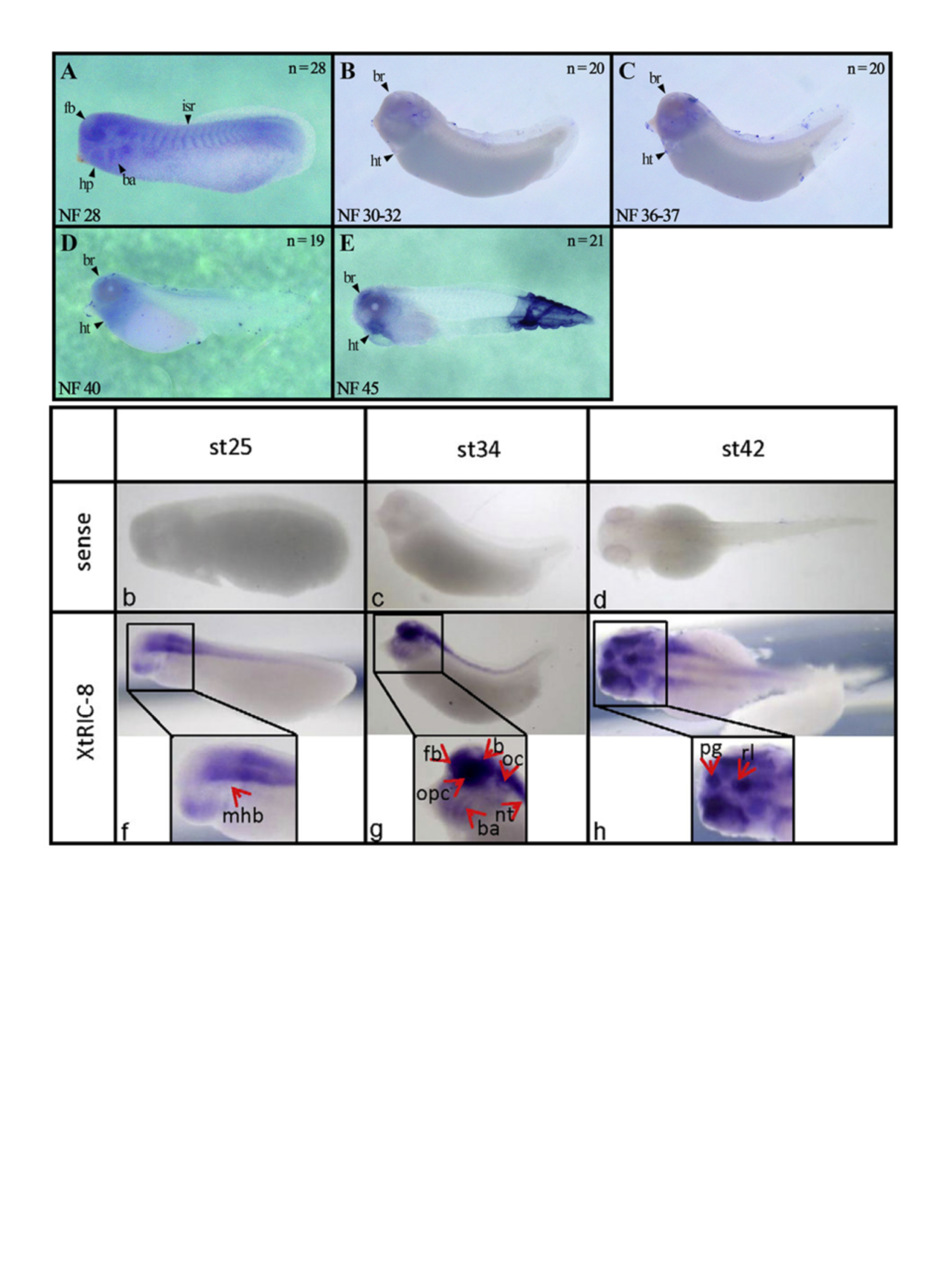

Supplement: Supplementary file 3 [file Image2.tiff]

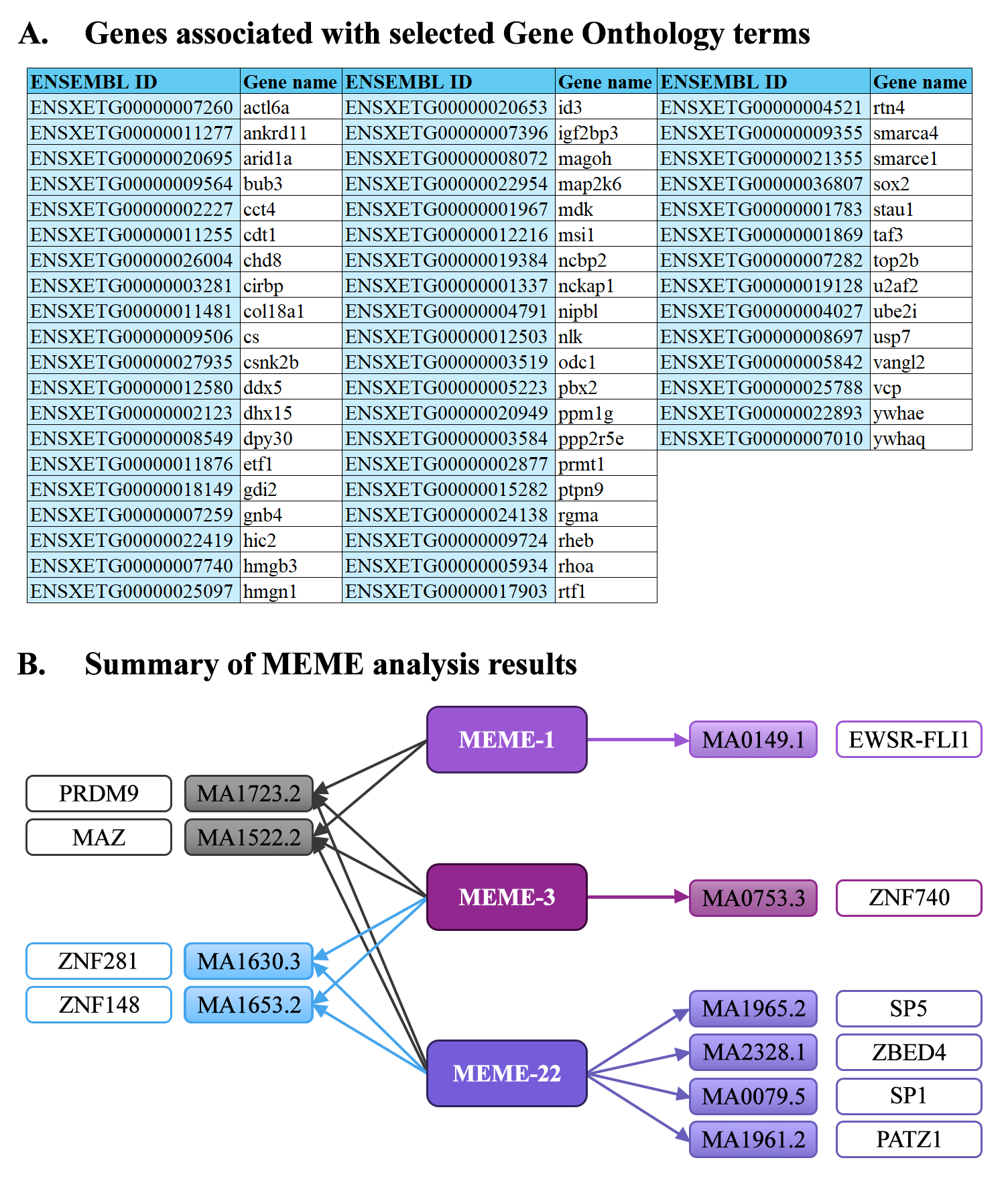

Supplement: Supplementary file 4 [file Image4.tiff]
